# Intestinal Microbiota and miRNA in IBD: A Narrative Review about Discoveries and Perspectives for the Future

**DOI:** 10.3390/ijms24087176

**Published:** 2023-04-13

**Authors:** Ellen Cristina Souza de Oliveira, Ana Elisa Valencise Quaglio, Daniéla Oliveira Magro, Luiz Claudio Di Stasi, Ligia Yukie Sassaki

**Affiliations:** 1 Department of Internal Medicine, Medical School, Sao Paulo State University (UNESP), Campus Botucatu, Sao Paulo CEP 18618-970, Brazil; 2 Laboratory of Phytomedicines, Pharmacology and Biotechnology (PhytoPharmaTec), Department of Biophysics and Pharmacology, Institute of Biosciences, Sao Paulo State University (UNESP), Campus Botucatu, Sao Paulo CEP 18618-689, Brazil; 3 Department of Surgery, Faculty of Medical Sciences, State University of Campinas (UNICAMP), Campinas, Sao Paulo CEP 13083-970, Brazil

**Keywords:** intestinal microbiota, microRNA, dysbiosis, ulcerative colitis, Crohn’s disease, inflammatory bowel

## Abstract

Inflammatory bowel disease (IBD) includes Crohn’s disease (CD) and ulcerative colitis (UC) and comprises a chronic gastrointestinal tract disorder characterized by hyperactive and dysregulated immune responses to environmental factors, including gut microbiota and dietary components. An imbalance of the intestinal microbiota may contribute to the development and/or worsening of the inflammatory process. MicroRNAs (miRNAs) have been associated with various physiological processes, such as cell development and proliferation, apoptosis, and cancer. In addition, they play an important role in inflammatory processes, acting in the regulation of pro- and anti-inflammatory pathways. Differences in the profiles of miRNAs may represent a useful tool in the diagnosis of UC and CD and as a prognostic marker in both diseases. The relationship between miRNAs and the intestinal microbiota is not completely elucidated, but recently this topic has gained prominence and has become the target of several studies that demonstrate the role of miRNAs in the modulation of the intestinal microbiota and induction of dysbiosis; the microbiota, in turn, can regulate the expression of miRNAs and, consequently, alter the intestinal homeostasis. Therefore, this review aims to describe the interaction between the intestinal microbiota and miRNAs in IBD, recent discoveries, and perspectives for the future.

## 1. Introduction

Inflammatory bowel disease (IBD) comprises a chronic gastrointestinal tract disorder characterized by hyperactive and dysregulated autoimmune responses and increased intestinal permeability related to environmental factors, including gut microbiota and dietary components, which includes Crohn’s disease (CD) and ulcerative colitis (UC), which differ in their pathophysiological and clinical characteristics [1,2].

The current incidence of CD has increased by 11% compared to the incidence three decades ago [3,4]. The etiology is unknown, and genetic, immunological, and environmental factors contribute to the risk of disease onset and progression. A cure remains elusive, and the efficient management of CD requires a multidisciplinary and interprofessional approach [4,5]. The disease can extend to all segments of the gastrointestinal tract, most commonly the terminal ileum and colon. Inflammation is typically segmental, asymmetrical, and transmural, but over time complications such as strictures, fistulas, or abscesses will develop in half of the patients who often require surgery [4,6,7]. Patients with CD frequently suffer from malnutrition and psychological issues and may have to live with a stoma, which could cause significant morbidity and impact the patients’ quality of life [4,7]. Current therapeutic strategies aim to prevent disease-related complications and interrupt the disease’s recurrence process by prolonging the remission period, whereas personalized medicine and the treat-to-target approach have been the most effective strategies adopted for the control of the inflammatory process [4,7].

UC is a chronic inflammatory condition that causes inflammation of the colon, manifested by continuous lesions and superficial inflammation, which can lead to erosions, ulcers, and bloody diarrhea [8]. The disease is characterized by a relapsing and remitting course, and curative medical therapy is not yet available. Patients with mild or moderate activity are usually unremarkable, apart from blood on rectal examination, whereas patients with a severe attack may exhibit fever, tachycardia, weight loss, abdominal tenderness, abdominal distension, and reduced bowel sounds [8,9].

The management of CD and UC has evolved from the mere treatment of symptoms and induction of clinical remission to more stringent outcomes, including the maintenance of steroid-free remission, the reduction in the number of hospitalizations and surgeries, mucosal and histological healing, improvement in patient-reported outcome, such as the patients’ quality of life [7,8,9,10,11], as well as the control of the risk factors associated with the development of colorectal cancer (CRC). The CRC risk is associated with the duration, extent, severity, and persistence of inflammatory activity [8,12], and it is estimated that CRC can account for up to 10% of deaths in patients with IBD [13].

As the etiology of IBD is not fully understood, it is believed that the interaction among genetic, immunological, and environmental factors, such as intestinal microbiota, can trigger the disease. Chronic gut dysbiosis has also been associated with autoimmune diseases such as eczema, asthma, celiac disease, and type-1 diabetes, as well as with diseases related to the consumption of an unbalanced diet, increased inactivity, age, obesity, type 2 diabetes, metabolic and cardiovascular diseases [14,15], liver disorders such as non-alcoholic liver steatosis [16], cancers such as colorectal cancer, and psychological diseases such as depression, anxiety, autism and Alzheimer’s disease [17,18,19].

During inflammation in IBD, oxidative stress promotes an increase in pathogenic bacteria at the expense of beneficial bacteria. This can cause an imbalance in the intestinal microbiota, potentially making it a useful biomarker and predictor for stratifying patients with IBD [20,21,22]. Further, microbiota-modulating therapies such as diet, fecal microbiota transplantation, pre- and probiotics, symbiotics, and antibiotics have been studied as potential therapies, thereby demonstrating the importance of intestinal microbiota in IBD patients [22,23,24,25,26].

miRNAs are potential disease markers that have been studied in recent years. miRNAs have been involved in the pathogenesis of IBD and their role has been studied both as a diagnostic biomarker and also as therapeutic targets [27,28]. miRNAs may represent a useful tool in the differentiation between UC and CD, besides being adopted as biomarkers of disease activity, of response to therapy as well as the potential to be used as prognostic markers of disease severity and the presence of complications such as stenosing, penetrating disease and CRC.

Both the intestinal microbiota and miRNAs have been the target of recent studies aimed at gaining an appreciation of their roles and relationship with IBD [29,30,31,32]. Considering that intestinal microbiota and miRNAs are both strongly related to IBD, understanding the role of each in the IBD process can provide vital information that could result in the development of more effective and accurate diagnostic tools and target treatments for patients with IBD. Therefore, this review aims to sed light on the current knowledge regarding intestinal microbiota and miRNAs, and their interaction with IBD, which has yet to be fully elucidated. Through highlighting recent discoveries, this review intends to provide insights into future research perspectives in this area.

## 2. Intestinal Microbiota and Inflammatory Bowel Disease

The intestinal microbiota in healthy individuals is known to provide countless benefits to the host, related to nutrition, metabolism, the immune system, and protection against pathogens [33]. More than 1000 species of bacteria reside in the human gastrointestinal tract, and it is estimated that the collective genome of these intestinal microorganisms contains approximately 100 times more genes than the human genome itself [34,35]. The human intestinal microbiota is composed mainly of bacteria, but also includes methanogenic archaea, viruses, fungi, yeasts, and protozoa [36].

Bacteria in the human intestine belong to six main phyla: Firmicutes, Bacteroidetes, Verrucomicrobia, Fusobacteria, Actinobacteria, and Proteobacteria, of which approximately 90% that compose the intestinal microbiota and colonize the gastrointestinal tract are members of the phyla Firmicutes and Bacteroidetes [33,36,37]. The phylum Firmicutes is diverse and comprises gram-positive bacteria with more than 200 different genera of strains, mainly *Ruminicoccus*, *Clostridium* and *Lactobacillus*, and butyrate-producing species, such as those belonging to the genera *Eubacterium*, *Faecalibacterium prausnitzii,* and *Roseburia*. The second most abundant phylum, Bacteroidetes, comprises gram-negative bacteria with approximately 20 genera, mainly *Bacteroides*, *Prevotella*, and *Xylanibacter*, which degrade a variety of complex glycans. The phylum Verrucomicrobia includes only one representative species, namely *Akkermansia muciniphila*, which is abundant in the gastrointestinal tract, and has attracted considerable interest in studies involving microbiota and IBD [38,39,40]. In the Fusobacteria phylum, a gram-negative anaerobic bacillus (*Fusobacterium nucleatum*) has a symbiotic relationship with its host but could also cause opportunistic infections and has recently garnered attention in several studies on the role of the microbiome in colorectal cancer [41,42]. The phylum Actinobacteria includes *Collinsella* and *Bifidobacterium*, while the common phylum Proteobacteria includes *Escherichia* and *Desulfovibrio* genera.

The diversity of the composition of the intestinal microbiota is related to the cultural and dietary habits in different regions of the world, including dwelling in rural or urban areas, climate, and pollution. The intestinal microbiota diversity also can be explained by the colonization and stabilization of an ecosystem in an existing niche that arises dependent on genetics, diet, age, and lifestyle [14]. The combination of specific diets, such as the concurrent use of prebiotics and probiotics, can have synergistic effects that promote modulation of the intestinal microbiota and protect the tissue from inflammation [43]. A recent study conducted with people from Canada and Ireland also showed that geographic location contributes for most of the microbiota variance, followed by previous surgical resection, alcohol consumption, use of supplements, age, gender, and drugs such as proton pump inhibitors, in IBD patients compared to controls [44]. The influence of geographic location on microbiota variance can be attributed to factors such as ethnicity and lifestyle, as highlighted in previous studies.

The human host for these intestinal bacteria provides a nutrient-rich environment, while the microbiota provides essential functions that humans cannot perform. Among these functions, the gut microbiota participates in the digestion of complex polysaccharides present in the host’s diet, which are important for the formation of monosaccharides and short-chain fatty acids (SCFAs), such as propionate, butyrate, and acetate that represent an important source of energy for the human body. SCFAs affect the proliferation, differentiation, and modulation of gene expression in mammalian colon epithelial cells [36], maintain the intestinal epithelial cell barrier and intestinal homeostasis [45], produce essential vitamins in the intestinal lumen, including vitamin K, vitamin B12, biotin, folate, thiamine, riboflavin and pyridoxine [46], regulate lipid and glucose metabolism through the composition of bile acid produced by the liver of the host [47], which induces regulatory mechanisms to maintain the balance between the intestinal mucosa and its immune system, resulting in the tolerance of harmless bacteria and activation of the immune system against pathogenic bacteria [48].

Through these symbiotic interactions, the intestinal microbiota co-evolved with the human species, becoming necessary to maintain the health of the host. The balance established in these interactions is largely preserved even in the face of sudden changes in the intestinal microbiota, as seen after antibiotic treatments, acute intestinal infections, changes in diet, or other external factors that affect the intestinal microbiota diversity, uniformity, relative abundance of species, and remodel the microbiota itself [49,50]. However, the loss of this balance results in unfavorable changes in the diversity and function of the intestinal microbiota, which can trigger an imbalance between beneficial and pathogenic bacteria in the intestine, termed dysbiosis, which impacts the interaction between the microbiota, the host, and their immune system.

The intestinal barrier, comprising intestinal epithelial cells, innate immune cells, intraepithelial lymphocytes, and the mucus layer, provides the first physical and chemical barrier against intestinal bacteria, pathogens, and food antigens [51]. Among the components of the physical intestinal barrier, the tight junctions (TJs) constitute the major determining constituent. Located close to the apical portion of the lateral membrane of epithelial cells, TJs connect to adjacent intestinal epithelial cells and are associated with cytoplasmic actin and myosin networks, which regulate intestinal permeability [51,52]. Disrupted TJs result in systemic and intestinal inflammation, leading to the dysregulation of both interactions, cytoskeletal rearrangements of intestinal epithelial cells, and increasing intestinal permeability [51,52]. These changes may favor the development of opportunistic infections, such as the overgrowth of *Enterobacteriaceae* or *Streptococcaceae*, followed by low-grade inflammation of the mucosa, increased intestinal permeability, local or systemic morphological and functional changes, due to the presence of pathogen microbial gene products [15,53], which can harm human health and favor the development of chronic diseases, such as IBD.

On the other hand, in IBD patients, the by-products of the host’s inflammatory response become environmental stressors, favoring bacterial growth [1,54]. This disturbance results in oxidative stress for the host and the intestinal microbiota, leading to intestinal dysbiosis with reduced representation of Firmicutes and Bacteroidetes species [55], and favoring the proliferation of facultative anaerobic *Enterobacteriaceae* and a particular group of *Escherichia coli* called adherent-invasive *E. coli* (AIEC) [54,56,57,58], which are frequently seen in patients with IBD and are believed to trigger the worsening of symptoms of the disease [59]. One property of these strains is the ability to adhere to and invade intestinal epithelial cells. Moreover, they can replicate within macrophages, inducing autophagic neutrophil cell death, and stimulate the production of reactive oxygen species (ROS), which contributes to intestinal inflammation [60,61,62,63]. However, it is unclear whether AIEC bacteria trigger intestinal inflammation leading to IBD or whether they serve as an aggravating factor for the disease [60].

During inflammation, the high concentrations of oxygen make the environment toxic to the obligate anaerobes, which leads to the reduction in the intestinal mucus layer, favoring the imbalance of the intestinal microbiota [20]. Metabolomic studies have found that the abundance of sphingomyelin, a class of plasma membrane-associated lipids produced by host and specific bacteria, such as *Bacteroides*, was low in patients with IBD [20,64,65]. When the abundance of sphingomyelin in the body decreased, the inflammatory response tended to rise [20,64,65].

Among the bacteria that constitute the intestinal microbiota, *F. prausnitzii* has been related to the pathogenesis of IBD [20,66,67,68]. Low level of *F. prausnitzii* in stool was related to disease relapse in CD patients [68]. It has also been shown that microbial anti-inflammatory molecule, a special protein produced by *F. prausnitzii*, can resist inflammation, and inhibits the nuclear factor-kappa B (NF-κB) pathway in several intestinal epithelial cell lines [68]. In fact, a study that used models in vitro (cellular models) and in vivo (2,4,6-trinitrobenzene sulfonic acid (TNBS)-induced colitis in mice) demonstrated the anti-inflammatory effects of *F. prausnitzi* through secretion of metabolites able to block NF-κB activation and interleukin (IL)-8 production, as well as the induction of anti-inflammatory cytokine secretion, such as IL-10 [69,70], suggesting the use of *F. prausnitzi* as a promising candidate probiotic agent in CD treatment.

Although the imbalance of the intestinal microbiota plays an important role in the etiology of IBD, the imbalance between beneficial and pathogenic bacteria alone does not cause the disease [20]. Some genes associated with IBD are known to be involved in mediating host responses to the intestinal microbiota, highlighting the possibility that the intestinal microbiota is implicated in the pathogenesis of IBD [33,71,72]. Several studies demonstrated that the composition of the intestinal microbiota in IBD is altered compared to that of healthy individuals [73,74,75,76]. Patients with IBD have fewer bacteria with protective properties (*Bifidobacterium species*, *Bacteroides*, and *Clostridium* Groups IV and XIVA with *F. prausnitzii* and *Roseburia species*) and more bacteria with pro-inflammatory properties (*Veillonellaceae*, *Pasteurellacaea*, *E. coli* (adherent/invasive), and *Fusobacteriaceae*) [55] (Table 1). Analysis of metagenomic sequencing data demonstrated that both CD and UC gut microbiota exhibit important changes compared to healthy gut microbiota [38]. Patients with active CD possess an altered microbial community compared with that of healthy individuals, and with patients with inactive CD; displaying an enrichment of *Ruminococcus gnavus* and *Escherichia* spp. and a reduction in Firmicutes [44]. These changes may be related to an increase in vascular and paracellular permeability [5,58,77]. On the other hand, patients with UC had a microbiota moderately different from healthy individuals [35,73,74,76].

Alterations in fecal concentrations of microbial metabolites have also been reported in IBD patients with a reduction in medium- and SCFAs, dysregulation of bile acid derivatives, and tryptophan metabolites [78,79]. SCFAs affect the differentiation and expansion of Treg cells and the growth of epithelial cells, known to play a pivotal role in maintaining intestinal homeostasis [79,80]. An increase in sulphate-reducing bacteria, such as *Disulfovibrio piger*, has also been described, resulting in higher hydrogen-sulphide that may harm intestinal epithelial cells and induce mucosal inflammation [81].

Despite the finding of dysbiosis in patients with CD and UC, studies aiming at modulating the intestinal microbiota through restrictive diets, fiber-based products, or the use of probiotics, have not reached definitive conclusions, and the indication of the use of prebiotics, probiotics, or symbiotics is reserved for specific cases. Recent research has observed the potential of polysaccharide-based hydrogels in modulating the intestinal microbiota, as they can be fermented by the intestinal microbiota to produce SFCAs. This prebiotic effect promotes the growth and viability of beneficial bacteria, resulting in enhanced stability and improved health outcomes. Given their ability to potentially prevent and/or treat chronic diseases, such as IBD, these hydrogels represent a promising avenue of research in this field [82]. However, a meta-analysis evaluated dietary interventions such as a high-fiber, low-refined carbohydrate diet, microparticle restriction, symptom-guided diet, restrictive or organic diets, and a low-calcium diet to induce remission or other outcomes such as quality of life, need for surgery, or disease progression, and concluded that the effects of dietary interventions on CD and UC are uncertain and more studies are necessary [83].

Data on the benefits of using probiotics in inducing remission in CD are inconclusive [84,85], but there is evidence of benefits in inducing disease remission in UC [85]. In addition, the use of a mixture of probiotics appears to be superior to using a simple strain [85], as demonstrated by a meta-analysis including 1049 patients and comparing VSL#3 (De Simone Formulation) with placebo [86]. The authors observed a higher remission rate and lower relapse rate in the VSL#3 (De Simone Formulation) group [86]. Moreover, *E. coli* Nissle 1917 shows comparable efficacy and safety to mesalazine [86]. Since the number of patients included in these studies was small, no definite conclusions can be made, and better designed and larger studies are needed.

Fecal microbiota transplantation is the most drastic way of modulating a person’s intestinal microbiota, showing promising results in IBD. A recent published systematic review and meta-analysis, which include sixty studies, showed the benefits of fecal microbiota transplantation in clinical remission and response for UC and CD [87]. The overall clinical remission and response rates were 37% and 53.8%, respectively, added to a prevalence of 29% of adverse events. The clinical remission rate was higher for the use of capsules (66.5%) compared to frozen fecal material (44.2%) or the use of fresh fecal material (29.1%). Universal donors showed better remission rates (45.1%) compared to relatives or acquaintances donors (24.8%). Patients with Crohn’s disease presented a higher remission rate (47.6%), whereas patients with UC showed a remission rate of 35.0%. The authors concluded that the use of frozen fecal material from universal donors may be related to a higher rate of clinical remission and response, especially in CD patients [87].

An innovative study carried out with pregnant women with IBD and their offspring showed a significant association of maternal microbiota composition during pregnancy and the microbiota of the offspring [88], highlighting the importance of environmental factors in the risk of developing IBD. The authors observed an altered bacterial composition and lower diversity in mothers with IBD and their babies, compared to those of babies born to controls, characterized by lower diversity and an enrichment in *Gammaproteobacteria* and a depletion of Bacteroidetes. Germ-free mice inoculated with the microbiota of pregnant patients with IBD, or their babies, showed an imbalanced immune and reduced microbial diversity when compared to the controls, suggesting that maternal IBD can impact the development of the immune system of the offspring [88]. Considering the central role of the gut microbiome in IBD pathogenesis, the results obtained assisted, in part, to explain the residual familiar risk of IBD in the offspring beyond the established shared genetic risk [88]. Further, the modulation of the microbiota of patients with IBD during pregnancy can impact the development of a healthier microbiota in their babies, thus decreasing the risk of the offspring developing IBD during their lifetime. However, further studies are required to confirm these findings and to clarify the risk-benefits of this intervention to the microbiome and to the immune system of pregnant women with IBD and their children.

In line with these data, a recent study evaluated pregnant women with IBD and their offspring up to the first year of life regarding the levels of fecal calprotectin, a surrogate marker of intestinal inflammation [89]. Due to the immuno-modulating function of fecal calprotectin, which may be involved in immune education in early life, and proper immune tolerance to commensal bacteria, the fecal calprotectin levels were correlated with the bacterial abundance in both mothers and babies [89]. Fecal calprotectin levels of pregnant patients with IBD were significantly higher than in control mothers. Moreover, fecal calprotectin levels were higher in babies whose mothers had active disease during pregnancy, when compared to babies born to mothers with inactive IBD [89]. These data suggest that disease activity might further contribute to mucosal inflammation in the baby, which could put them at a higher risk of developing IBD or other immune diseases later in life, due to a reduced ability to achieve optimal mucosal immunity or establish intestinal barrier function [89].

Further, it has been observed that fungi coexisting with bacteria within the intestine can also play an important role in IBD. Studies showed an increased level of *Candida albicans* in patients with IBD [90,91]. *Saccharomyces cerevisiae* represent an important component of the normal fungal microbiota, and it has been shown to reduce colitis induced by adherent-invasive *E. coli*, a species of bacteria associated with ileal CD in CEACAM6-expressing mice [92]. The role of the fungi microbiota may differ in UC and CD. In patients with UC, the biodiversity in bacteria and fungi is associated with new interactions that may be involved in the inflammatory process [90]. In contrast, patients with CD are characterized by disrupted connections between bacterial and fungal microbiota [90].

Therefore, it has been observed that several factors can influence the composition of the intestinal microbiota promoting dysbiosis. A direct relationship between dysbiosis and IBD has not yet been established in humans and it is still unclear whether inflammation caused by IBD can trigger dysbiosis or the reverse process occurs, i.e., whether the dysbiosis precedes the disease. All data that have been discovered and identified thus far indicate that there is still considerable research required to better understand this relationship.

## 3. MicroRNAs in Inflammatory Bowel Disease

miRNAs are a group of small (18–24 nucleotides), single-stranded, non-coding RNA molecules that can act as potent negative regulators in gene expression [93,94]. Each miRNA can target hundreds of mRNAs within a given cell type, and a single mRNA is often the target of multiple miRNAs. Thus, miRNAs contribute to the regulation of >30% of protein-coding genes [95]. Several biological processes are regulated by miRNAs, including cell survival, differentiation, proliferation, apoptosis, cell cycle control, and homeostasis; additionally, specific miRNAs regulate the differentiation of intestinal epithelial cells [93,94].

miRNAs have been extensively studied in multiple types of cancer and have been reported as regulators of tumor suppressors and oncogenes [95]. Although most studies are focused on their aforementioned role, the impact on autoimmune diseases and especially IBD is not fully investigated (Table 2). It has been suggested that the critical function of these small RNAs is to contribute to the establishment of immunological homeostasis at mucosal sites [93].

The first study reporting miRNA alterations in IBD patients revealed 11 different expressed miRNAs in patients with UC vs. controls. The miRNAs miR-16, miR-21, miR-23a, miR-24, miR-29a, miR-126, miR-195, and let-7f were increased, whereas miR-192, miR-375, and miR-422b were reduced [96]. Subsequently, several studies have been conducted with the aim of characterizing such alterations in the expression of miRNAs [97,98,99] and, consequently, several of these miRNAs have been suggested as potential biomarkers for CD or UC both in colonic tissues and non-invasive samples such as blood and feces [99,100,101,102].

The distinct miRNA expression was described in tissues from different intestinal regions in patients with active ileal or colonic CD [97]. Three miRNAs were increased (miR-31, miR-215, miR-22), and one miRNA (miR-19b) was decreased in the terminal ileum compared to those in the colon, supporting the likelihood that miRNAs influence different inflammation-related gene expression in each IBD subtype [97].

Another study reported miR-223 as a potential biomarker in the serum of patients with IBD [102]. Patients with IBD had significantly increased serum levels of miR-223 to controls, showing a positive correlation with disease activity in patients with CD and UC. Moreover, miR-223 showed a better disease activity correlation in patients with CD compared to erythrocyte sedimentation rate and high-sensitivity C-reactive protein [102]. In another study, the circulating miR-320a levels were strongly correlated with endoscopic disease activity in patients with CD and UC, highlighting its potential as a non-invasive biomarker in monitoring the control of the inflammatory process [103].

In addition to their potential role in monitoring disease activity, whether in clinical [102], biochemical [102], or endoscopic activity [103], miRNA can also be used as predictors of response to therapy. When evaluating patients with severe UC, Morilla et al. [104] identified 15 miRNAs associated with the response to corticosteroids, 6 miRNAs associated with the response to infliximab, and 4 associated with the response to cyclosporine, in those patients unresponsive to initial corticosteroid therapy, thereby highlighting the role of miRNA as a predictor of response to therapy in IBD. In other study including children with IBD (CD: 17 and UC: 2) who received prednisone or infliximab, miR-146a, miR-320a, and miR-146b decreased with both drugs, correlating to the control of the inflammatory process, and miR-486 showed a significant change in response to prednisone but not to infliximab [105].

**Table 2:** The main microRNA involved in Inflammatory Bowel Disease patients.

miRNA	In IBD	Target	References
miR10a	Decrease	Inhibit NOD2	[106]
miR-16	Increase in UC	T-cell sub-types	[96]
miR-19b	Decrease in CD		[97]
miR-21	Increase in UC	T-cell sub-types	[96]
miR-22	Increase in CD	Th17 cell	[97]
miR-23a	Increase in UC		[96]
miR-24	Increase in UC		[96]
miR-29	Decrease IL-12 and IL-23 in CD	Through activation of NOD2	[107,108]
miR-29a	Increase in UC	NOD2	[108]
miR-31	Increase in CD		[97]
miR-107	Decrease	IL-23p19 (a subunit of IL-23)	[109]
miR-126	Increase	Regulates VCAM-1	[96,110,111]
miR-143/145	Decrease	Inhibit IGFBP5 (regulate IGF pathway in intestinal epithelial regeneration)	[32,112]
miR-146a	Increase	TNF-α	[105]
miR-146b	Increase	TNF-α	[105]
miR-150	Increase intestinal permeability	c-Myb	[32,113]
miR-155	Increase in UC	SOCS1	[114]
miR-192	Decrease in UC	MIP-2α	[96]
miR-195	Increase in UC		[96]
miR-215	Increase in CD	MIP-2α	[97]
miR-223	Increase	Claudin-8 (a TJ-integral protein)	[102]
miR-320a	Increase		[103,105]
miR-375	Decrease in UC	inhibit KLF5 (antagonist of the goblet cell–differentiation factor KLF4)	[32,96]
miR-422b	Decrease in UC		[96]
miR-486	Increase		[105]
let-7f	Increase in UC	T-cell sub-types	[96]

Relationship of main microRNA with decreased or increased activity in Inflammatory Bowel Disease (IBD). CD: Crohn’s disease; IGFBP5: insulin-like growth factor-binding protein 5; KLF5/4: Kruppel-like factor 5 and 4; MIP-2α: macrophage inhibitory peptide; NOD2: nucleotide-binding oligomerization domain 2; SOCS1: Suppressor of Cytokine Signaling 1; Th17: T helper 17; TJ: tight junction; TNF-α: tumor necrosis factor alpha; UC: ulcerative colitis; VCAM-1: Vascular Cell Adhesion Molecule-1.

In addition to their role as markers of inflammatory activity, miRNAs may be the actual therapeutic target in the future. Some identified miRNAs act on the same inflammatory pathways as some medications approved for the treatment of IBD. miR-29 has been observed to comprise a family of miRNAs with the potential to decrease levels of IL-23 [108,110], such as ustekinumab, an antibody that inhibits IL12/23, indicated in moderate to severe CD. miR-126 inhibits the leukocyte adhesion to endothelial cells through the regulation of Vascular Cell Adhesion Molecule-1 (VCAM-1) [110,111], the same mechanism of action of the vedolizumab, also indicated for the treatment of moderate to severe IBD. The miR-155 Suppressor of Cytokine Signaling 1 (SOCS1) targets a regulatory protein of the Janus Kinase (JAK) signaling pathway [114], mimicking the use of JAK inhibitors currently available for UC treatment. A review published by Moein et al. [115] clarified the relationship among various miRNAs and the mechanisms involved in the pathogenesis of IBD, including modulations of the inflammatory response through dendritic cells, macrophages, neutrophils, natural killer cells and T cells, dysregulation of TJs, formation of the mucous barrier, and regulation of apoptosis. The elucidation of the role of miRNAs in the inflammatory cascade opens new innovative perspectives for the treatment of IBD, for example, by providing the enhancement of miRNAs that act by inhibiting the inflammatory response (using RNA mimics) or inhibiting the miRNAs that act to perpetuate the inflammatory response (using antagonists of miRNAs).

Changes in the profiles of miRNAs could represent a useful tool in the differentiation of UC and CD, providing important information about the pathophysiology of each disease, prognosis, and response to therapy. Moreover, the identification of the dysregulated miRNA may represent new targets for new therapies focusing on modulation of the inflammatory process via miRNA regulation. Future studies are required for better characterization of the miRNA profile in IBD patients and to clarify the role of miRNAs in triggering and maintaining the inflammatory process in IBD patients and their applications in clinical practice.

## 4. Interaction between microRNAs and Microbiota in Inflammatory Bowel Disease

**Figure 1:**
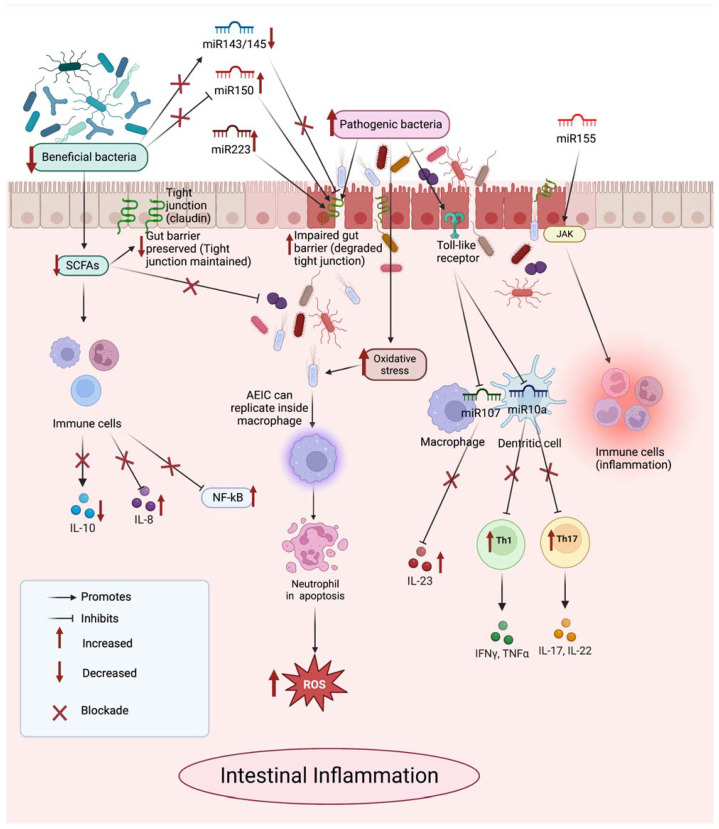
Interaction between microRNA and microbiota in Inflammatory Bowel Disease. AEIC: adherent-invasive *E. coli*; IFN-γ: interferon-gamma; IL: interleukin; NF-κB: nuclear factor-kappa B; ROS: reactive oxygen species; SCFAs: short-chain fatty acids; TNF-α: tumor necrosis factor alpha.

Recently, the role of miRNAs and their interactions with the host and its microbiota have been gaining prominence and becoming the target of several studies that demonstrate the participation of miRNAs in the modulation of the intestinal microbiota and induction of dysbiosis, whereas the intestinal microbiota, in turn, can regulate the expression of miRNAs and consequently alter intestinal homeostasis [29,30,116,117,118] (Figure 1).

### 4.1. MicroRNA and Its Role in the Modulation of Microbiota

Endogenous or exogenous miRNAs might actively interact with the microbiota and impact bacterial gene expression [119]. Although the mechanism of this interaction remains unknown, it has been noted that small RNAs present in bacteria function similarly to miRNAs [119], which may provide insight into how this interaction is established.

Other studies show that miRNAs can enter bacteria, such as *F. nucleatum* and *E. coli*, bind to DNA, and specifically regulate bacterial gene transcripts, affecting the growth of the intestinal microbiota [120,121] and establishing another possible form of interaction between miRNAs and bacteria. In fact, a study using fecal samples from patients with IBD demonstrated that abnormal expression of miRNAs has different effects on the proliferative activity of intestinal microorganisms including *F. nucleatum*, *E. coli*, and segmental filamentous bacteria, which can cause an imbalance of the intestinal microbiota, leading to the occurrence of diseases [122].

One study showed that host fecal miRNAs can regulate microbial fitness and gene expression [120]. Interestingly, this study revealed that mice deficient in the miRNA-generating protein had dysbiosis and were more susceptible to colitis than wild-type mice, suggesting that the microbial community may be affected by miRNAs [31,120]. The regulation of host gene expression is one means of communication between the gut microbiota and the host through the manipulation of host miRNA expression, such as fecal extracellular miRNAs [120,123]. This regulation has been related to the release of miRNA in extracellular vesicles, which are taken up by microbes and may affect microbial growth [120,123]. It was observed that intestinal epithelial cells are the major sources of extracellular fecal miRNAs, due to their ability to secrete exosome-like vesicles [120,123]. Corroborating these findings, one study demonstrated that mice with deletion of miR-149-3p exhibited changes in their microbiota and greater intensity of intestinal inflammation induced by dextran sulfate sodium (DSS) [112]. These results suggest that miRNAs may play an important role in regulating the intestinal microbiota.

However, it is important to highlight that the miRNAs are involved in many important gene regulation processes, not just uptake into bacterial cells, which could also impact dysbiosis by other indirect means [113,122]. A study in an experimental model of colitis demonstrated that mice with miR-223 deficiency exhibited more intense intestinal inflammation due to the increase in pro-inflammatory cytokines [106]. miR-223 plays a critical role in regulating the differentiation and function of intestinal macrophages and dendritic cells, and consequently, it regulates the production of pro-inflammatory cytokines, helping to maintain intestinal homeostasis [106]. This regulation may indirectly affect the composition of the microbiota present in this environment.

Therefore, we can infer that miRNAs can serve as potential biomarkers of dysbiosis, contributing to the diagnosis and/or treatment of IBD.

### 4.2. Microbiota and Its Role in the Expression of microRNA

One study sought to determine whether the microbiota modulated the expression of miRNAs in the host. For this, germ-free mice were colonized with the intestinal microbiota from pathogen-free mice, and comparison of the miRNA expression profile revealed dysregulated miRNA expression in the ileum and colon of these mice, demonstrating that expression of host miRNAs is dysregulated in response to the intestinal microbiota colonization [124].

Reinforcing this data, a recent study using different probiotics (*L. fermentum* CECT5716, *L. salivarius* CECT5713, *E. coli* Nissle 1917, *S. boulardii* CNCMI-745) in a dinitrobenzene sulfonic acid (DNBS) model on intestinal inflammation in mice demonstrated the beneficial effects of probiotics associated with their ability to modify the intestinal microbiota and the immune response, which could be achieved at a posttranscriptional level by modifying the expression of miRNAs, such as restoring the expression of miR-143/145, which is highly expressed in the normal colon tissue [109], and reduce the expression of miR-150, which may be responsible for the increased intestinal permeability seen in intestinal inflammation [32,125]. In another experimental model of DSS-induced colitis in mice, the use of two probiotics (*L. fermentum* and *L. salivarius*) improved dysbiosis and reduced the expression of miR-155, miR-223, miR-150, and miR-143, which act on the permeability of the intestinal barrier and on the exacerbated response of pro-inflammatory cytokines, resulting in improvement of intestinal inflammation [126]. Similarly, the use of the probiotic *S. boulardii* in the same experimental model of colitis in mice showed an improvement in dysbiosis and a reduction in the expression of miR-155 and miR-223 [127].

Another recent study using fecal microbiota transplantation in humans was performed with two groups, an allogenic (received feces from lean healthy donor) and an autologous (control = received own feces). This study observed a significant correlation between the fecal miRNA expression and microbiota composition for both groups, evidencing an involvement of the gut microbiota in regulating intestinal miRNA expression [30].

Pathogenic bacteria, such as *Helicobacter pylori*, AIEC, among others, can also alter the expression of miRNAs as a strategy to survive in host cells and cause infections and inflammation [128]. This can result in the alteration of the intestinal microbiota and the immune system, contributing to the evolution of various diseases, such as IBD. In fact, a study conducted with an experimental mice model of intestinal inflammation induced by means of AIEC infection showed an increase in the expression of miR-30C and miR-130A via NF-κB, providing a greater number of intracellular AIEC, and consequently, a greater inflammatory response [129]. In a recent study utilizing in vivo and in vitro models of IBD and CRC, Enterotoxigenic *Bacteroides fragilis* (ETBF), a bacterium strongly associated with these diseases was found to negatively regulate miR-149-3p, which plays an important role in inhibiting tumor cells, after treatment with ETBF [130].

Thus, we observed that several studies have highlighted that the intestinal microbiota can affect the host’s miRNA expression, and one of the suggested ways to explain this action is the production of bacterial metabolites short-chain fatty acids (SCFAs), such as butyrate [131,132,133]. Other studies suggest that negative regulation of miRNA expression, such as miR-10a (suppresses Th1 and Th17 cell response, via Nucleotide-binding Oligomerization Domain 2 (NOD2)) and miR107 (regulates IL23 production, via IL-23p19), in response to a high level of commensal bacterial stimulation, can occur through the participation of toll-like receptors and Myd88-dependent pathways [134,135,136]. However, so far, the exact mechanism by which intestinal microbiota influence microRNA expression is unclear.

### 4.3. Perspectives on the Interaction of microRNA and Microbiota in Inflammatory Bowel Disease

**Figure 2:**
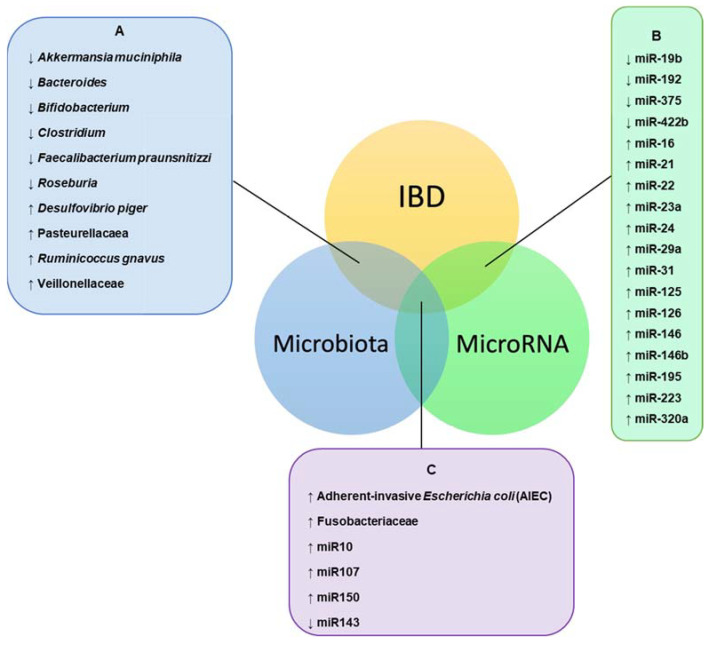
Complex relationship of intestinal microbiota and microRNA in Inflammatory Bowel Disease (IBD). The intersection between IBD, microbiota, and miRNA represent the points of influence between the disease and the profiles of intestinal bacteria and miRNA. A = the main bacteria involved in dysbiosis in IBD; B = the main miRNAs involved with IBD; C = complex interaction between intestinal bacteria and miRNA profiles and the disease, which can offer new perspectives regarding evolution and control of the IBD, still underexplored. IBD = inflammatory bowel disease; ↓ = reduced bacteria and reduced miRNA in IBD; ↑ = increased bacteria and increased miRNA in IBD.

In recent years, the role of miRNAs in the interaction with the host and its microbiota has been increasingly explored, aiming to clarify and/or find new ways of diagnosing and treating chronic diseases, such as IBD. The approach to the complex relationship between intestinal microbiota and miRNAs in IBD was described in a recent study [113]; however, there is still much to be explored within this theme (Figure 2).

We have come across studies that demonstrate the role of beneficial bacteria in regulating mRNA expression, thereby ameliorating the symptoms of IBD. However, there are also reports indicating the ability of pathogenic bacteria to modulate miRNA expression, leading to dysbiosis and aggravation of the inflammatory response and worsening the symptoms of IBD. On the other hand, miRNA has been found to participate directly or indirectly participate in the modulation of the intestinal microbiota, which also influences the IBD condition. MicroRNA directly regulate the barrier function of the intestinal epithelium and the absorption of bacterial metabolites to maintain intestinal homeostasis, and indirectly remodel the intestinal microbiota [137].

According to the findings of this review, we can infer that microRNAs play an important role in communication between the intestinal microbiota and the host, which may favor the maintenance of intestinal homeostasis and the prevention of diseases, such as IBD and CRC. Thus, miRNAs could function as therapeutic tools that could act on the host and/or the intestinal microbiota and benefit patients with IBD. We can also consider the use of diets supplemented with prebiotics or probiotics, which may help in modulating the intestinal microbiota and regulating the expression of miRNAs, with the goal of improving the intestinal inflammatory process in patients with IBD. However, all these perspectives require further studies and research to better understand and apply them in the future.

## 5. Conclusions

Several studies have highlighted the important role of dysbiosis in the development of IBD and how strategies aimed at maintaining the balance of the intestinal microbiota can be useful in the treatment and/or prevention of the development of the disease. miRNAs have also become the target of studies that have contributed to the understanding of the disease and brought new perspectives to the diagnosis and treatment of patients with IBD. However, future studies are required to facilitate a better understanding of the complex interaction between the intestinal microbiota and the miRNAs involved in IBD pathogenesis, to elucidate their role in diagnosis and as therapeutic targets, and to consolidate their applications in clinical practice.

## Figures and Tables

**Table 1:** The main bacteria involved in dysbiosis in Inflammatory Bowel Disease patients.

Family/*Species*	In IBD	Protective Action on Intestinal Tissue	Pathogenic Action on Intestinal Tissue	References
*Escherichia coli* (adherent-invasive)	Increase		It triggers the worsening of IBD symptoms	[60]
*Bacteroides*	Decrease	Obligatory anaerobe, regulate the intestinal inflammation		[20]
*Faecalibacterium prausnitzii*	Decrease	It resists inflammation and can inhibit the NF-κB and induced the production of IL-10		[68,70]
*Ruminococcus* *gnavus*	Increase		It affects the balance of the intestinal mucus layer, which may increase the intestinal permeability	[58]
*Bifidobacterium*	Decrease	It is important producer of SCFA		[55]
*Lactobacillus*	Decrease	Limited biosynthetic abilities and ferment sugars, generating lactic acid as major product		[55]
*Clostridium*	Decrease	It is indispensable regulator of intestinal homeostasis		[55]
*Akkermansia* *muciniphila*	Decrease	it is a mucus-colonizing bacterium, studies have demonstrated an anti- inflammatory effect		[38,55]
*Roseburia*	Decrease	SCFA producer with anti- inflammatory effects		[55]
*Fusobacterium* *nucleatum*	Increase		Obligatory anaerobic, but can cause opportunistic infections after ileocecal resection	[55]
*Disulfovibrio piger*	Increase		It harms intestinal epithelial cells and induces mucosal inflammation	[55]

Relationship of the bacteria with protective and pathogenic action in Inflammatory Bowel Disease (IBD). In IBD, bacteria with protective action are decreased while pathogenic bacteria are increased, resulting in dysbiosis. NF-κB: nuclear factor κB; SCFA: short-chain fatty acids.

## Data Availability

No new data were created or analyzed in this study. Data sharing is not applicable to this article.

## Abbreviations

AIEC	Adherent-invasive *E. coli*
CD	Crohn’s Disease
CRC	Colorectal Cancer
DNBS	Dinitrobenzene Sulfonic Acid
DSS	Dextran Sulfate Sodium
ETBF	Enterotoxigenic *Bacteroides fragilis*
JAK	Janus Kinase
IBD	Inflammatory Bowel Disease
IFN-γ	Interferon-gamma
IGFBP5	Insulin-like growth factor-binding protein 5
IL	Interleukin
KLF5/4	Kruppel-like factor 5 and 4
MIP-2α	Macrophage Inhibitory Peptide 2 alpha
miRNA	MicroRNA
NF-κB	Nuclear Factor-kappa B
NOD2	Nucleotide-binding Oligomerization Domain 2
ROS	Reactive Oxygen Species
SCFAs	Short-chain Fatty Acids
SOCS1	Suppressor of Cytokine Signaling 1
Th17	T helper 17
TJs	Tight junctions
TNBS	Trinitrobenzene Sulfonic Acid
TNF-α	Tumor Necrosis Factor alpha
UC	Ulcerative Colitis
VCAM-1	Vascular Cell Adhesion Molecule 1

## References

[1] Hills R.D., Pontefract B.A., Mishcon H.R., Black C.A., Sutton S.C., Theberge C.R. (2019). Gut microbiome: Profound implications for diet and disease. Nutrients.

[2] Oligschlaeger Y., Yadati T., Houben T., Condello Oliván C.M., Shiri-Sverdlov R. (2019). Inflammatory Bowel Disease: A Stressed “Gut/Feeling”. Cells.

[3] Zhao M., Gönczi L., Lakatos P.L., Burisch J. (2021). The Burden of Inflammatory Bowel Disease in Europe in 2020. J. Crohn’s Colitis.

[4] Adamina M., Bonovas S., Raine T., Spinelli A., Warusavitarne J., Armuzzi A., Bachmann O., Bager P., Biancone L., Bokemeyer B. (2020). ECCO Guidelines on Therapeutics in Crohn’s Disease: Surgical Treatment. J. Crohn’s Colitis.

[5] Roda G., Chien N.S., Kotze P.G., Argollo M., Panaccione R., Spinelli A., Kaser A., Peyrin-Biroulet L., Danese S. (2020). Crohn’s disease. Nat. Rev. Dis. Prim..

[6] Torres J., Mehandru S., Colombel J.F., Peyrin-Biroulet L. (2017). Crohn’s disease. Lancet.

[7] Torres J., Bonovas S., Doherty G., Kucharzik T., Gisbert J.P., Raine T., Adamina M., Armuzzi A., Bachmann O., Bager P. (2020). ECCO Guidelines on Therapeutics in Crohn’s Disease: Medical treatment. J. Crohn’s Colitis.

[8] Magro F., Gionchetti P., Eliakim R., Ardizzone S., Armuzzi A., Barreiro-de Acosta M., Burisch J., Gecse K.B., Hart A.L., Hindryckx P. (2017). Third European evidence-based consensus on diagnosis and management of ulcerative colitis. Part 1: Definitions, diagnosis, extra-intestinal manifestations, pregnancy, cancer surveillance, surgery, and ileo-anal pouch disorders. J. Crohn’s Colitis.

[9] Harbord M., Eliakim R., Bettenworth D., Karmiris K., Katsanos K., Kopylov U., Kucharzik T., Molnár T., Raine T., Sebastian S. (2017). Third European evidence-based consensus on diagnosis and management of ulcerative colitis. Part 2: Current management. J. Crohn’s Colitis.

[10] Ordás I., Eckmann L., Talamini M., Baumgart D.C., Sandborn W.J. (2012). Ulcerative colitis. Lancet.

[11] Danese S., Roda G., Peyrin-Biroulet L. (2020). Evolving therapeutic goals in ulcerative colitis: Towards disease clearance. Nat. Rev. Gastroenterol. Hepatol..

[12] Quaglio A.E.V., Grillo T.G., Oliveira E.C.S.D., Stasi L.C.D., Sassaki L.Y. (2022). Gut microbiota, inflammatory bowel disease and colorectal cancer. World J. Gastroenterol..

[13] Nadeem M.S., Kumar V., Al-Abbasi F.A., Kamal M.A., Anwar F. (2020). Risk of colorectal cancer in inflammatory bowel diseases. Semin. Cancer Biol..

[14] Harmsen H.J.M., de Goffau M.C., Schwiertz A. (2016). The Human Gut Microbiota. Microbiota of the Human Body—Implications in Healthy and Disease.

[15] Lynch S.V., Pedersen O. (2016). The human intestinal microbiome in health and disease. N. Engl. J. Med..

[16] Milosevic I., Vujovic A., Barac A., Djelic M., Korac M., Spurnic A.R., Gmizic I., Stevanovic O., Djordjevic V., Levic N. (2019). Gut-liver axis, gut microbiota, and its modulation in the management of liver diseases: A review of the literature. Int. J. Mol. Sci..

[17] Altveş S., Yildiz H.K., Vural H.C. (2020). Interaction of the microbiota with the human body in health and diseases. Biosci. Microb. Food Health.

[18] Treisman G.J., Floch M., Ringel Y., Walker W.A. (2017). The Role of the Brain–Gut–Microbiome in Mental Health and Mental Disorders. The Microbiota in Gastrointestinal Pathophysiology.

[19] Dai Z., Coker O.O., Nakatsu G., Wu W.K.K., Zhao L., Chen Z., Chan F.K.L., Kristiansen K., Sung J.J.Y., Wong S.H. (2018). Multi-cohort analysis of colorectal cancer metagenome identified altered bacteria across populations and universal bacterial markers. Microbiome.

[20] Zheng L., Wen X.-L. (2021). Gut microbiota and inflammatory bowel disease: The current status and perspectives. World. J. Clin. Cases.

[21] Guo X., Huang C., Xu J., Xu H., Liu L., Zhao H., Wang J., Huang W., Peng W., Chen Y. (2022). Gut Microbiota Is a Potential Biomarker in Inflammatory Bowel Disease. Front. Nutr..

[22] Zhang Y., Li Y., Ren X., Zhang X., Wu Z., Liu L. (2023). The positive correlation of antioxidant activity and prebiotic effect about oat phenolic compounds. Food Chem..

[23] Zhou J., Li M., Chen Q., Li X., Chen L., Dong Z., Zhu W., Yang Y., Liu Z., Chen Q. (2022). Programmable probiotics modulate inflammation and gut microbiota for inflammatory bowel disease treatment after effective oral delivery. Nat. Commun..

[24] Yang Y., Zheng X., Wang Y., Tan X., Zou H., Feng S., Zhang H., Zhang Z., He J., Cui B. (2022). Human Fecal Microbiota Transplantation Reduces the Susceptibility to Dextran Sulfate Sodium-Induced Germ-Free Mouse Colitis. Front. Immunol..

[25] Dixit K., Chaudhari D., Dhotre D., Shouche Y., Saroj S. (2021). Restoration of dysbiotic human gut microbiome for homeostasis. Life Sci..

[26] Martyniak A., Medyńska-Przęczek A., Wędrychowicz A., Skoczeń S., Tomasik P.J. (2021). Prebiotics, probiotics, synbiotics, paraprobiotics and postbiotic compounds in IBD. Biomolecules.

[27] Feng Y., Zhang Y., Zhou D., Chen G., Li N. (2019). MicroRNAs, intestinal inflammatory and tumor. Bioorganic Med. Chem. Lett..

[28] James J.P., Riis L.B., Malham M., Høgdall E., Langholz E., Nielsen B.S. (2020). MicroRNA biomarkers in IBD-differential diagnosis and prediction of colitis-associated cancer. Int. J. Mol. Sci..

[29] Johnston D.G.W., Williams M.A., Thaiss C.A., Cabrera-Rubio R., Raverdeau M., McEntee C., Cotter P.D., Elinav E., O’Neill L.A.J., Corr S.C. (2018). Loss of microRNA-21 influences the gut microbiota, causing reduced susceptibility in a murine model of colitis. J. Crohn’s Colitis.

[30] Wortelboer K., Bakker G.J., Winkelmeijer M., Van Riel N., Levin E., Nieuwdorp M., Herrema H., Davids M. (2022). Fecal microbiota transplantation as tool to study the interrelation between microbiota composition and miRNA expression. Microbiol. Res..

[31] Viennois E., Chassaing B., Tahsin A., Pujada A., Wang L., Gewirtz A.T., Merlin D. (2019). Host-derived fecal microRNAs can indicate gut microbiota healthiness and ability to induce inflammation. Theranostics.

[32] Algieri F., Garrido-Mesa J., Vezza T., Rodríguez-Sojo M.J., Rodríguez-Cabezas M.E., Olivares M., Garcia F., Gálvez J., Morón R., Rodríguez-Nogales A. (2020). Intestinal anti-inflammatory effects of probiotics in DNBS-colitis via modulation of gut microbiota and microRNAs. Eur. J. Nutr..

[33] Nishida A., Inoue R., Inatomi O., Bamba S., Naito Y., Andoh A. (2018). Gut microbiota in the pathogenesis of inflammatory bowel disease. Clin. J. Gastroenterol..

[34] Adak A., Khan M.R. (2019). An insight into gut microbiota and its functionalities. Cell Mol. Life Sci..

[35] Qin J., Li R., Raes J., Arumugam M., Burgdorf K.S., Manichanh C., Nielsen T., Pons N., Levenez F., Yamada T. (2010). A human gut microbial gene catalogue established by metagenomic sequencing. Nature.

[36] Sánchez-Tapia M., Tovar A.R., Torres N. (2019). Diet as Regulator of Gut Microbiota and its Role in Health and Disease. Arch. Med. Res..

[37] Eckburg P.B., Bik E.M., Bernstein C.N., Purdom E., Sargent M., Gill S.R., Gill S.R., Nelson K.E., Relman D.A. (2005). Diversity of the Human Intestinal Microbial Flora. Science.

[38] Zhai R., Xue X., Zhang L., Yang X., Zhao L., Zhang C. (2019). Strain-specific anti-inflammatory properties of two *Akkermansia muciniphila* strains on chronic colitis in mice. Front. Cell Infect. Microbiol..

[39] Zhai Q., Feng S., Arjan N., Chen W. (2019). A next generation probiotic, *Akkermansia muciniphila*. Crit. Rev. Food Sci. Nutr..

[40] Zhang T., Li Q., Cheng L., Buch H., Zhang F. (2019). *Akkermansia muciniphila* is a promising probiotic. Microb. Biotechnol..

[41] Wu J., Li Q., Fu X. (2019). *Fusobacterium nucleatum* Contributes to the Carcinogenesis of Colorectal Cancer by Inducing Inflammation and Suppressing Host Immunity. Transl. Oncol..

[42] Berbert L., Santos A., Magro D.O., Guadagnini D., Assalin H.B., Lourenço L.H., Martinez C.A.R., Saad M.J.A., Coy C.S.R. (2022). Metagenomics analysis reveals universal signatures of the intestinal microbiota in colorectal cancer, regardless of regional differences. Braz. J. Med. Biol. Res..

[43] Shinde T., Vemuri R., Shastri S., Perera A.P., Gondalia S.V., Beale D.J., Karpe A.V., Eri R., Stanley R. (2020). Modulating the microbiome and immune responses using whole plant fibre in synbiotic combination with fibre-digesting probiotic attenuates chronic colonic inflammation in spontaneous colitic mice model of IBD. Nutrients.

[44] Clooney A.G., Eckenberger J., Laserna-Mendieta E., Sexton K.A., Bernstein M.T., Vagianos K., Sargent M., Ryan F.J., Moran C., Sheehan D. (2020). Ranking microbiome variance in inflammatory bowel disease: A large longitudinal intercontinental study. Gut.

[45] Soderholm A.T., Pedicord V.A. (2019). Intestinal epithelial cells: At the interface of the microbiota and mucosal immunity. Immunology.

[46] Rowland I., Gibson G., Heinken A., Scott K., Swann J., Thiele I., Tuohy K. (2018). Gut microbiota functions: Metabolism of nutrients and other food components. Eur. J. Nutr..

[47] Ramírez-Pérez O., Cruz-Ramón V., Chinchilla-López P., Méndez-Sánchez N. (2017). The Role of the Gut Microbiota in Bile Acid Metabolism. Ann. Hepatol..

[48] Dollé L., Tran H.Q., Etienne-Mesmin L., Chassaing B. (2016). Policing of gut microbiota by the adaptive immune system. BMC Med..

[49] Drago L., Valentina C., Fabio P. (2019). Gut microbiota, dysbiosis and colon lavage. Dig. Liver Dis..

[50] Rothschild D., Weissbrod O., Barkan E., Kurilshikov A., Korem T., Zeevi D., Costea P.I., Godneva A., Kalka I.N., Bar N. (2018). Environment dominates over host genetics in shaping human gut microbiota. Nature.

[51] Peterson L.W., Artis D. (2014). Intestinal epithelial cells: Regulators of barrier function and immune homeostasis. Nat. Rev. Immunol..

[52] Suzuki T. (2020). Regulation of the intestinal barrier by nutrients: The role of tight junctions. Anim. Sci. J..

[53] Stecher B., Maier L., Hardt W.D. (2013). “Blooming” in the gut: How dysbiosis might contribute to pathogen evolution. Nat. Rev. Microbiol..

[54] Winter S.E., Bäumler A.J. (2014). Dysbiosis in the inflamed intestine: Chance favors the prepared microbe. Gut Microb..

[55] Caenepeel C., Tabib N.S.S., Vieira-Silva S., Vermeire S. (2020). Review article: How the intestinal microbiota may reflect disease activity and influence therapeutic outcome in inflammatory bowel disease. Aliment. Pharm. Ther..

[56] Martinez-Medina M., Garcia-Gil L.J. (2014). *Escherichia coli* in chronic inflammatory bowel diseases: An update on adherent invasive *Escherichia coli* pathogenicity. World J. Gastrointest. Pathophysiol..

[57] Balmus I., Ciobica A., Trifan A., Stanciu C. (2016). The implications of oxidative stress and antioxidant therapies in Inflammatory Bowel Disease: Clinical aspects and animal models. Saudi J. Gastroenterol..

[58] Hall A.B., Yassour M., Sauk J., Garner A., Jiang X., Arthur T., Lagoudas G.K., Vatanen T., Fornelos N., Wilson R. (2017). A novel *Ruminococcus gnavus* clade enriched in inflammatory bowel disease patients. Genome Med..

[59] Honda K., Littman D.R. (2012). The Microbiome in Infectious Disease and Inflammation. Annu. Rev. Immunol..

[60] Palmela C., Chevarin C., Xu Z., Torres J., Sevrin G., Hirten R., Barnich N., Ng S.C., Colombel J.F. (2018). Adherent-invasive *Escherichia coli* in inflammatory bowel disease. Gut.

[61] Fang X., Monk J.M., Nurk S., Akseshina M., Zhu Q., Gemmell C., Gianetto-Hill C., Leung N., Szubin R., Sanders J. (2018). Metagenomics-based, strain-level analysis of *Escherichia coli* from a time-series of microbiome samples from a Crohn’s disease patient. Front. Microbiol..

[62] Chargui A., Cesaro A., Mimouna S., Fareh M., Brest P., Naquet P., Darfeuille-Michaud A., Hébuterne X., Mograbi B., Vouret-Craviari V. (2012). Subversion of Autophagy in Adherent Invasive *Escherichia coli*-Infected Neutrophils Induces Inflammation and Cell Death. PLoS ONE.

[63] Vong L., Yeung C.W., Pinnell L.J., Sherman P.M. (2016). Adherent-invasive *Escherichia coli* Exacerbates Antibiotic-associated Intestinal Dysbiosis and Neutrophil Extracellular Trap Activation. Inflamm. Bowel. Dis..

[64] Ding N.S., McDonald J.A.K., Perdones-Montero A., Rees D.N., Adegbola S.O., Misra R., Hendy P., Penez L., Marchesi J.R., Holmes E. (2020). Metabonomics and the gut microbiome associated with primary response to anti-TNF therapy in Crohn’s disease. J. Crohn’s Colitis.

[65] Fischbeck A., Leucht K., Frey-Wagner I., Bentz S., Pesch T., Kellermeier S., Krebs M., Fried M., Rogler G., Hausmann M. (2011). Sphingomyelin induces cathepsin D-mediated apoptosis in intestinal epithelial cells and increases inflammation in DSS colitis. Gut.

[66] Zhao H., Xu H., Chen S., He J., Zhou Y., Nie Y. (2021). Systematic review and meta-analysis of the role of *Faecalibacterium prausnitzii* alteration in inflammatory bowel disease. J. Gastroenterol. Hepatol..

[67] Lopez-Siles M., Enrich-Capó N., Aldeguer X., Sabat-Mir M., Duncan S.H., Garcia-Gil L.J., Martinez-Medina M. (2018). Alterations in the Abundance and Co-occurrence of *Akkermansia muciniphila* and *Faecalibacterium prausnitzii* in the Colonic Mucosa of Inflammatory Bowel Disease Subjects. Front. Cell Infect. Microbiol..

[68] Quévrain E., Maubert M.A., Michon C., Chain F., Marquant R., Tailhades J., Miquel S., Carlier L., Bermúdez-Humarán L.G., Pigneur B. (2016). Identification of an anti-inflammatory protein from *Faecalibacterium prausnitzii*, a commensal bacterium deficient in Crohn’s disease. Gut.

[69] Sokol H., Pigneur B., Watterlot L., Lakhdari O., Bermúdez-Humarán L.G., Gratadoux J.-J., Blugeon S., Bridonneau C., Furet J.P., Corthier G. (2008). *Faecalibacterium prausnitzii* is an anti-inflammatory commensal bacterium identified by gut microbiota analysis of Crohn disease patients. Proc. Natl. Acad. Sci. USA.

[70] Ananthakrishnan A.N. (2020). Microbiome-based biomarkers for ibd. Inflamm. Bowel Dis..

[71] Liu J.Z., Van Sommeren S., Huang H.N.S.C., Alberts R., Takahashi A., Ripke S., Lee J.C., Jostins L., Shah T. (2015). Association analyses identify 38 susceptibility loci for inflammatory bowel disease and highlight shared genetic risk across populations. Nat. Genet..

[72] Sheehan D., Moran C., Shanahan F. (2015). The microbiota in inflammatory bowel disease. J. Gastroenterol..

[73] Magro D.O., Santos A., Guadagnini D., de Godoy F.M., Silva S.H.M., Lemos W.J.F., Virtulo N., Torriani S., Pinheiro L.V., Martinez C.A.R. (2019). Remission in Crohn’s disease is accompanied by alterations in the gut microbiota and mucins production. Sci. Rep..

[74] Weng Y.J., Gan H.Y., Li X., Huang Y., Li Z.C., Deng H.M., Chen S.Z., Zhou Y., Wang L.S., Han Y.P. (2019). Correlation of diet, microbiota and metabolite networks in inflammatory bowel disease. J. Dig. Dis..

[75] Sun Y., Li L., Xia Y., Li W., Wang K., Wang L., Miao Y., Ma S. (2019). The gut microbiota heterogeneity and assembly changes associated with the IBD. Sci. Rep..

[76] Zhou Y., Xu Z.Z., He Y., Yang Y., Liu L., Lin Q., Nie Y., Li M., Zhi F., Liu S. (2018). Gut Microbiota Offers Universal Biomarkers across Ethnicity in Inflammatory Bowel Disease Diagnosis and Infliximab Response Prediction. mSystems.

[77] Henke M.T., Kenny D.J., Cassilly C.D., Vlamakis H., Xavier R.J., Clardy J. (2019). *Ruminococcus gnavus*, a member of the human gut microbiome associated with Crohn’s disease, produces an inflammatory polysaccharide. Proc. Natl. Acad. Sci. USA.

[78] Marchesi J.R., Holmes E., Khan F., Kochhar S., Scanlan P., Shanahan F., Wilson I.D., Wang Y. (2007). Rapid and noninvasive metabonomic characterization of inflammatory bowel disease. J. Proteom. Res..

[79] Lavelle A., Sokol H. (2020). Gut microbiota-derived metabolites as key actors in inflammatory bowel disease. Nat. Rev. Gastroenterol. Hepatol..

[80] Smith P.M., Howitt M.R., Panikov N., Michaud M., Gallini C.A., Bohlooly Y.M., Glickman J.N., Garrett W.S. (2013). The microbial metabolites, short-chain fatty acids, regulate colonic T reg cell homeostasis. Science.

[81] Kushkevych I., Coufalová M., Vítězová M., Rittmann S.K.-M.R. (2020). Sulfate-Reducing Bacteria of the Oral Cavity and Their Relation with Periodontitis—Recent Advances. J. Clin. Med..

[82] Zhang Y., Dong L., Liu L., Wu Z., Pan D., Liu L. (2022). Recent Advances of Stimuli-Responsive Polysaccharide Hydrogels in Delivery Systems: A Review. J. Agric. Food Chem..

[83] Limketkai B.N., Iheozor-Ejiofor Z., Gjuladin-Hellon T., Parian A., Matarese L.E., Bracewell K., MacDonald J.K., Gordon M., Mullin G.E. (2019). Dietary interventions for induction and maintenance of remission in inflammatory bowel disease. Cochrane Database Syst. Rev..

[84] Limketkai B.N., Akobeng A.K., Gordon M., Adepoju A.A. (2020). Probiotics for induction of remission in Crohn’s disease. Cochrane Database Syst. Rev..

[85] Pabón-Carrasco M., Ramirez-Baena L., Vilar-Palomo S., Castro-Méndez A., Martos-García R., Rodríguez-Gallego I. (2020). Probiotics as a coadjuvant factor in active or quiescent inflammatory bowel disease of adults—A meta-analytical study. Nutrients.

[86] Jia K., Tong X., Wang R., Song X. (2018). The clinical effects of probiotics for inflammatory bowel disease: A meta-analysis. Medicine.

[87] Caldeira L.F., Borba H.H., Tonin F.S., Wiens A., Fernandez-Llimos F., Pontarolo R. (2020). Fecal microbiota transplantation in inflammatory bowel disease patients: A systematic review and meta-analysis. PLoS ONE.

[88] Torres J., Hu J., Seki A., Eisele C., Nair N., Huang R., Tarassishin L., Jharap B., Cote-Daigneault J., Mao Q. (2020). Infants born to mothers with IBD present with altered gut microbiome that transfers abnormalities of the adaptive immune system to germ-free mice. Gut.

[89] Kim E.S., Tarassishin L., Eisele C., Barre A., Nair N., Rendon A., Hawkins K., Debebe A., White S., Thjømøe A. (2021). Longitudinal Changes in Fecal Calprotectin Levels Among Pregnant Women With and Without Inflammatory Bowel Disease and Their Babies. Gastroenterology.

[90] Sokol H., Leducq V., Aschard H., Pham H.P., Jegou S., Landman C., Cohen D., Liguori G., Bourrier A., Nion-Larmurier I. (2017). Fungal microbiota dysbiosis in IBD. Gut.

[91] Lam S., Zuo T., Ho M., Chan F.K.L., Chan P.K.S., Ng S.C. (2019). Review article: Fungal alterations in inflammatory bowel diseases. Aliment. Pharm. Ther..

[92] Sivignon A., De Vallée A., Barnich N., Denizot J., Darcha C., Pignède G., Vandekerckove P., Darfeuille-Michaud A. (2015). *Saccharomyces cerevisiae* CNCMI-3856 prevents colitis induced by AIEC bacteria in the transgenic mouse model mimicking Crohn’s disease. Inflamm. Bowel Dis..

[93] Archanioti P., Gazouli M., Theodoropoulos G., Vaiopoulou A., Nikiteas N. (2011). Micro-RNAs as regulators and possible diagnostic bio-markers in inflammatory bowel disease. J. Crohn’s Colitis.

[94] Coskun M., Bjerrum J.T., Seidelin J.B., Nielsen O.H. (2012). MicroRNAs in inflammatory bowel disease-pathogenesis, diagnostics and therapeutics. World. J. Gastroenterol..

[95] Dalal S.R., Kwon J.H. (2010). The role of microRNA in inflammatory bowel disease. Gastroenterol. Hepatol..

[96] Wu F., Zikusoka M., Trindade A., Dassopoulos T., Harris M.L., Bayless T.M., Brant S.R., Chakravarti S., Kwon J.H. (2008). MicroRNAs Are Differentially Expressed in Ulcerative Colitis and Alter Expression of Macrophage Inflammatory Peptide-2α. Gastroenterology.

[97] Wu F., Zhang S., Dassopoulos T., Harris M.L., Bayless T.M., Meltzer S.J., Brant S.R., Kwon J.H. (2010). Identification of microRNAs associated with ileal and colonic Crohn’s disease. Inflamm. Bowel Dis..

[98] Mohammadi A., Kelly O.B., Filice M., Kabakchiev B., Smith M.I., Silverberg M.S. (2018). Differential expression of microRNAs in peripheral blood mononuclear cells identifies autophagy and TGF-beta-related signatures aberrantly expressed in inflammatory bowel disease. J. Crohn’s Colitis.

[99] Masi L., Capobianco I., Magrì C., Marafini I., Petito V., Scaldaferri F. (2022). MicroRNAs as Innovative Biomarkers for Inflammatory Bowel Disease and Prediction of Colorectal Cancer. Int. J. Mol. Sci..

[100] Suri K., Bubier J.A., Wiles M.V., Shultz L.D., Amiji M.M., Hosur V. (2021). Role of microRNA in inflammatory bowel disease: Clinical evidence and the development of preclinical animal models. Cells.

[101] Schönauen K., Le N., Von Arnim U., Schulz C., Malfertheiner P., Link A. (2018). Circulating and fecal microRNAs as biomarkers for inflammatory bowel diseases. Inflamm. Bowel Dis..

[102] Wang H., Zhang S., Yu Q., Yang G., Guo J., Li M., Zeng Z., He Y., Chen B., Chen M. (2016). Circulating MicroRNA223 is a new biomarker for inflammatory bowel disease. Medicine.

[103] Cordes F., Demmig C., Bokemeyer A., Brückner M., Lenze F., Lenz P., Nowacki T., Tepasse P., Schmidt H.H., Schmidt M.A. (2020). MicroRNA-320a Monitors Intestinal Disease Activity in Patients With Inflammatory Bowel Disease. Clin. Transl. Gastroenterol..

[104] Morilla I., Uzzan M., Laharie D., Cazals-Hatem D., Denost Q., Daniel F., Belleannee G., Bouhnik Y., Wainrib G., Panis Y. (2019). Colonic MicroRNA Profiles, Identified by a Deep Learning Algorithm, That Predict Responses to Therapy of Patients with Acute Severe Ulcerative Colitis. Clin. Gastroenterol. Hepatol..

[105] Heier C.R., Fiorillo A.A., Chaisson E., Gordish-Dressman H., Hathout Y., Damsker J.M., Hoffman E.P., Conklin L.S. (2016). Identification of Pathway-Specific Serum Biomarkers of Response to Glucocorticoid and Infliximab Treatment in Children with Inflammatory Bowel Disease. Clin. Transl. Gastroenterol..

[106] Zhou H., Xiao J., Wu N., Liu C., Xu J., Liu F., Wu L. (2015). MicroRNA-223 Regulates the Differentiation and Function of Intestinal Dendritic Cells and Macrophages by Targeting C/EBPβ. Cell Rep..

[107] Bae H.J., Noh J.H., Kim J.K., Eun J.W., Jung K.H., Kim M.G., Chang Y.G., Shen Q., Kim S.J., Park W.S. (2014). MicroRNA-29c functions as a tumor suppressor by direct targeting oncogenic SIRT1 in hepatocellular carcinoma. Oncogene.

[108] Chapman C.G., Pekow J. (2015). The emerging role of miRNAs in inflammatory bowel disease: A review. Therap. Adv. Gastroenterol..

[109] Chivukula R.R., Shi G., Acharya A., Mills E.W., Zeitels L.R., Anandam J.L., Abdelnaby A.A., Balch G.C., Mansour J.C., Yopp A.C. (2014). An essential mesenchymal function for miR-143/145 in intestinal epithelial regeneration. Cell.

[110] Harris T.A., Yamakuchi M., Ferlito M., Mendell J.T., Lowenstein C.J. (2008). MicroRNA-126 regulates endothelial expression of vascular cell adhesion molecule 1. Proc. Natl. Acad. Sci. USA.

[111] Fedyk E.R., Wyant T., Yang L.L., Csizmadia V., Burke K., Yang H., Kadambi V.J. (2012). Exclusive antagonism of the α4β7 integrin by vedolizumab confirms the gut-selectivity of this pathway in primates. Inflamm. Bowel Dis..

[112] Feng Q., Li Y., Zhang H., Wang Z., Nie X., Yao D., Han L., Chen W.D., Wang Y.D. (2022). Deficiency of miRNA-149-3p shaped gut microbiota and enhanced dextran sulfate sodium-induced colitis. Mol. Ther.-Nucleic Acids.

[113] Casado-Bedmar M., Viennois E. (2022). MicroRNA and Gut Microbiota: Tiny but Mighty-Novel Insights into Their Cross-talk in Inflammatory Bowel Disease Pathogenesis and Therapeutics. J. Crohns Colitis.

[114] Pathak S., Grillo A.R., Scarpa M., Brun P., D’Incà R., Nai L., Banerjee A., Cavallo D., Barzon L., Palú G. (2015). MiR-155 modulates the inflammatory phenotype of intestinal myofibroblasts by targeting SOCS1 in ulcerative colitis. Exp. Mol. Med..

[115] Moein S., Vaghari-Tabari M., Qujeq D., Majidinia M., Nabavi S.M., Yousefi B. (2019). MiRNAs and inflammatory bowel disease: An interesting new story. J. Cell Physiol..

[116] Feng Q., Chen W.D., Wang Y.D. (2018). Gut microbiota: An integral moderator in health and disease. Front. Microbiol..

[117] Dongiovanni P., Meroni M., Longo M., Fargion S., Fracanzani A.L. (2018). MiRNA signature in NAFLD: A turning point for a non-invasive diagnosis. Int. J. Mol. Sci..

[118] Peck B.C.E., Mah A.T., Pitman W.A., Ding S., Lund P.K., Sethupathy P. (2017). Functional transcriptomics in diverse intestinal epithelial cell types reveals robust MicroRNA sensitivity in intestinal stem cells to microbial status. J. Biol. Chem..

[119] Yuan C., Steer J.C., Subramanian S. (2019). Host–MicroRNA–Microbiota Interactions in Colorectal Cancer. Genes.

[120] Liu S., Da Cunha A.P., Rezende R.M., Cialic R., Wei Z., Bry L., Comstock L.E., Gandhi R., Weiner H.L. (2016). The Host Shapes the Gut Microbiota via Fecal MicroRNA. Cell Host. Microbe.

[121] Yang Y., Jobin C. (2017). Novel Insights into Microbiome in Colitis and Colorectal Cancer. Curr. Opin. Gastroenterol..

[122] Ji Y., Li X., Zhu Y., Li N., Zhang N., Niu M. (2018). Faecal microRNA as a biomarker of the activity and prognosis of inflammatory bowel diseases. Biochem. Biophys. Res. Commun..

[123] Williams M.R., Stedtfeld R.D., Tiedje J.M., Hashsham S.A. (2017). MicroRNAs-based inter-domain communication between the host and members of the gut microbiome. Front. Microbiol..

[124] Dalmasso G., Nguyen H.T.T., Yan Y., Laroui H., Charania M.A., Ayyadurai S., Sitaraman S.V., Merlin D. (2011). Microbiota modulate host gene expression via micrornas. PLoS ONE.

[125] Wang S., Huang Y., Zhou C., Wu H., Zhao J., Wu L., Zhao M., Zhang F., Liu H. (2018). The role of autophagy and related microRNAs in inflammatory bowel disease. Gastroenterol. Res. Pract..

[126] Rodríguez-Nogales A., Algieri F., Garrido-Mesa J., Vezza T., Utrilla M.P., Chueca N., García F., Olivares M., Rodríguez-Cabezas M.E., Gálvez J. (2017). Differential intestinal anti-inflammatory effects of *Lactobacillus fermentum* and *Lactobacillus salivarius* in DSS mouse colitis: Impact on microRNAs expression and microbiota composition. Mol. Nutr. Food Res..

[127] Rodríguez-Nogales A., Algieri F., Garrido-Mesa J., Vezza T., Utrilla M.P., Chueca N., García F., Rodríguez-Cabezas M.E., Gálvez J. (2018). Intestinal anti-inflammatory effect of the probiotic *Saccharomyces boulardii* in DSS-induced colitis in mice: Impact on microRNAs expression and gut microbiota composition. J. Nutr. Biochem..

[128] Rad Z.R., Rad Z.R., Goudarzi H., Goudarzi M., Mahmoudi M., Sharahi J.Y., Hashemi A. (2021). MicroRNAs in the interaction between host–bacterial pathogens: A new perspective. J. Cell Physiol..

[129] Nguyen H.T.T., Dalmasso G., Müller S., Carrière J., Seibold F., Darfeuille-Michaud A. (2014). Crohn’s disease-associated adherent invasive *Escherichia coli* modulate levels of microRNAs in intestinal epithelial cells to reduce autophagy. Gastroenterology.

[130] Cao Y., Wang Z., Yan Y., Ji L., He J., Xuan B., Shen C., Ma Y., Jiang S., Ma D. (2021). Enterotoxigenic *Bacteroides fragilis* Promotes Intestinal Inflammation and Malignancy by Inhibiting Exosome-Packaged miR-149-3p. Gastroenterology.

[131] Belcheva A. (2017). MicroRNAs at the epicenter of intestinal homeostasis. BioEssays.

[132] Gasaly N., Hermoso M.A., Gotteland M. (2021). Butyrate and the fine-tuning of colonic homeostasis: Implication for inflammatory bowel diseases. Int. J. Mol. Sci..

[133] Du J., Zhang P., Luo J., Shen L., Zhang S., Gu H., He J., Wang L., Zhao X., Gam M. (2021). Dietary betaine prevents obesity through gut microbiota-drived microRNA-378a family. Gut Microb..

[134] Wu W., He C., Liu C., Cao A.T., Xue X., Evans-Marin H.L., Sun M., Fang L., Yao S., Pinchuk I.V. (2015). miR-10a inhibits dendritic cell activation and Th1/Th17 cell immune responses in IBD. Gut.

[135] Larsson E., Tremaroli V., Lee Y.S., Koren O., Nookaew I., Fricker A., Nielsen J., Ley R.E., Bäckhed F. (2012). Analysis of gut microbial regulation of host gene expression along the length of the gut and regulation of gut microbial ecology through MyD88. Gut.

[136] Xue X., Cao A.T., Cao X., Yao S., Carlson E.D., Soong L., Liu C.G., Liu X., Liu Z., Duck L.W. (2014). Downregulation of MicroRNA-107 in intestinal CD11c+ myeloid cells in response to microbiota and proinflammatory cytokines increases IL-23p19 expression. Eur. J. Immunol..

[137] Fan Y., Qin M., Zhu J., Chen X., Luo J., Chen T., Sun J., Zhang Y., Xi Q. (2022). MicroRNA sensing and regulating microbiota-host crosstalk via diet motivation. Crit. Rev. Food Sci. Nutr..

